# Flow cytometry-based functional selection of RNA interference triggers for efficient epi-allelic analysis of therapeutic targets

**DOI:** 10.1186/1472-6750-14-57

**Published:** 2014-06-21

**Authors:** David R Micklem, Magnus Blø, Petra Bergström, Erlend Hodneland, Crina Tiron, Torill Høiby, Christine Gjerdrum, Ola Hammarsten, James B Lorens

**Affiliations:** 1 Department of Biomedicine, University of Bergen, N-5009 Bergen, Norway; 2 BerGenBio A/S, N-5009, Bergen, Norway; 3 Department of Clinical Chemistry and Transfusion Medicine, Sahlgrenska University Hospital, S-413 45 Göteborg, Sweden

## Abstract

**Background:**

The dose-response relationship is a fundamental pharmacological parameter necessary to determine therapeutic thresholds. Epi-allelic hypomorphic analysis using RNA interference (RNAi) can similarly correlate target gene dosage with cellular phenotypes. This however requires a set of RNAi triggers empirically determined to attenuate target gene expression to different levels.

**Results:**

In order to improve our ability to incorporate epi-allelic analysis into target validation studies, we developed a novel flow cytometry-based functional screening approach (CellSelectRNAi) to achieve unbiased selection of shRNAs from high-coverage libraries that knockdown target gene expression to predetermined levels. Employing a Gaussian probability model we calculated that knockdown efficiency is inferred from shRNA sequence frequency profiles derived from sorted hypomorphic cell populations. We used this approach to generate a hypomorphic epi-allelic cell series of shRNAs to reveal a functional threshold for the tumor suppressor p53 in normal and transformed cells.

**Conclusion:**

The unbiased CellSelectRNAi flow cytometry-based functional screening approach readily provides an epi-allelic series of shRNAs for graded reduction of target gene expression and improved phenotypic validation.

## Background

Delineating gene function relies on the genetic principle established by decades of genetic mapping that mutations establish causality in phenotypes. *Trans*-dominant genetic effectors such as small molecule inhibitors and small interfering RNA (siRNA) that alter the expression and function of specific genes and proteins *in trans*, have dramatically enhanced our ability to correlate the extent of cell behavioral changes with the perturbation of gene expression and function. Pharmacological dose response studies correlate the degree of compound-induced inhibition of protein function with cellular responses. Similarly, hypomorphic alleles, mimicked by RNAi, have served as valuable tools for deciphering the roles of various genes in cell function [1,2]. Such epi-allelic hypomorphic analysis using different RNAi triggering sequences empirically determined to knockdown gene expression to distinct levels, provides a unique opportunity to correlate gene expression levels with phenotypic outcome [3]. Hence, epi-allelic analysis can provide a dose response profile for putative therapeutic targets in lieu of small molecule inhibitors.

In order to improve our ability to conduct epi-allelic analysis, we developed a robust flow cytometry-based functional screening approach to enable unbiased selection of shRNAs that generate graded knockdown of target gene expression to predetermined levels. This genetic dose-response can be used to validate candidate drug targets and predict *in vivo* responses to inhibitor compounds.

## Methods

### Cell culture

All cells were cultured in a humidified atmosphere at 37°C with 5% CO_2_. Phoenix A retroviral packaging cells (Dr. Garry Nolan, Stanford University, USA) and MDA-MB-231 cells (ATCC) were maintained in DMEM (Gibco) and F-12 K nutrient mixture (*Kaighn’s modification*) respectively, both supplemented with 10% fetal bovine serum (FBS) (EuroClone, Italy), 2 mM L-glutamine, 100 U/ml penicillin and 100 μg/ml streptomycin (all from Sigma-Aldrich) [4]. Leukemic cell line HL60 (gift from Dr. Bjørn T. Gjertsen; University of Bergen, Norway) were cultured in Iscoves medium containing 10% FBS, antibiotics and glutamine (Sigma-Aldrich) [5]. Primary human umbilical vein endothelial cells (HUVEC) (C2517A, Lonza, Switzerland) were maintained in the supplier’s recommended complete medium (EGM-2).

### Retroviral transductions

Phoenix A cells were transfected using the calcium phosphate method [4]. Approximately 30 h after transfection, the medium was changed to the appropriate growth medium for the cells to be infected. Infectious supernatant was harvested 48 h after transfection. Target cells were spin-infected in virus-containing supernatant supplemented with 5 μg/ml protamine sulfate for 90 min at 1200 × g, and then incubated overnight before being returned to regular growth medium. Infected cells were selected with 1 μg/ml puromycin for 48 h.

### Immunostaining and flow cytometry

The MDA-MB-231 cells were trypsinized using standard procedures and washed with 0.2% BSA/PBS. Cells were stained with anti-Axl antibody (MAB154; R&D Systems, USA) at a final concentration of 2 μg/ml in 0.2% BSA/PBS for 40 min. at room temperature. The cells were then washed twice in 0.2% BSA/PBS and incubated with secondary antibody (goat anti-mouse allophyocyanin-crosslinked; Molecular Probes, USA) at a final concentration of 0.2 μg/ml for 30 min. at room temperature in the dark. The cells were washed twice with 0.2% BSA/PBS and resuspended in 300 μl of 0.2% BSA/PBS before analysis on a FACSAria cell sorter (BD Biosciences, USA). Data analyses were carried out with FlowJo software (Tree Star, USA).

### Constructs

Functional shRNAs that were identified from the library screen, were recloned into a new vector. The shRNAs were expressed from a modified human U6 promoter in the LTR of the retroviral vectors RRI-Red/L087 (GenBank: EU424173). For sequence information on shAxl2, shAxl278, shAxl279, shAxl280 and shLuc see [2].

The retroviral expression vector pCSI expresses a puromycin-N-acetyl-transferase-EGFP and firefly luciferase cassette (Puro2A- GFP2ALuc2), with each open-reading frame separated by a linker encoding the 2A region (XXSGLRSGQLLNFDLLKLAGDVES- NPGP) from foot-and-mouth disease virus for stoichiometric co-translational polyprotein cleavage [6].

### Construction of the reporter gene vector

Reporter constructs were made by cloning the target cDNA (Axl: 2.9 kb EcoRI/BamHI fragment from IMAGE cDNA clone 5205825; p53: 1.5 kb PshA1/NotI fragment from pSVp53 (gift from B.T. Gjertsen) into the 3′-untranslated region of retroviral vector L101 (Complete sequence in Additional file 1: Figure S1).

### shRNA library construction

The construction of the Axl library has been described previously [2]. The p53 library (p53 cDNA kindly provided by BT Gjertsen, University of Bergen) was produced by a new method (see Additional file 2: Figure S2), generating a larger number of longer hairpins. p53 cDNA was fragmented with each of the following frequently-cutting enzymes: HpaII/Hin6I (mixed), AluI, DpnI, BsuRI, RsaI, HpyCH4V and blunt-ended with Klenow. The fragments in each digestion mix were then capped at each end by ligation with excess Hairpin oligo DK540 (PhoCTGCTGGATCCAGAGACTCCAGAATTGTGAGCGCTCACAATTCTGGAGATGAGCCTTCGGTCTCATCTCTGGATCCAGCAG). PvuII and an appropriate frequent-cutter enzyme were added to each mixture to inhibit unwanted side reactions. After overnight ligation, reactions were pooled, phenol extracted, chloroform extracted and size-selectively precipitated with 15% PEG 8000/0.1 M MgCl_2_/20 μg glycogen to get rid of excess DK540 [7,8] Hairpin structures comprising 27 nt of p53-derived sequence joined to hairpin oligo DK540 were released from each end of the capped fragments by digestion with EcoP15I, dephosphorylated with Antarctic Phosphatase (NEB) and cleaned up by phenol/chloroform extraction and ethanol precipitation. Following blunt-ending with Klenow the hairpin loops (apparent MW approx 80 bp) were run on a 2% agarose/5 mM sodium borate gel [9] and purified by electroelution onto GF/C paper (Whatman).

The hairpins were cloned and converted into hairpin expression cassettes in a single step: Dephosphorylated hairpins were ligated to NaeI/NarI-cut L267 pEntr-U6NaeIXcmI, a kanamycin resistant derivative of pDONR221 carrying a PacI-U6 promoter-Termination signal-HindIII cassette, and then cut with XcmI. The relatively small size of this vector compared to retroviral vector L087 simplifies the cloning and gives improved transformation efficiencies. The product of ligation contains a nick at the 5′ end of the hairpin due to the lack of 5′ phosphate. Extension from the free 3′OH at this nick with a strand-displacing polymerase (Klenow) opens out the hairpin structure into dsDNA and at the same time removes the single nucleotide 3′ XcmI overhang. The construct was then recircularised and electroporated into competent cells and plated. This primary library (many thousand colonies) was scraped up and plasmid DNA isolated. The size of the hairpin loop was reduced by digestion with GsuI, blunt-ending with Klenow, gel-purification, recircularisation and re-cloning. Finally, the complete U6:hairpin:termination signal cassette was transferred into retroviral vector L297, a slight modification of vector L087. Restriction enzymes and polymerases were from New England Biolabs or Fermentas.

### Isolation of p53 targeting shRNAs

To select shRNAs that target human p53 mRNA from the approximately 100 unique shRNA sequences, we prepared virus supernatants from the p53 shRNA library and control shLuc vectors as described above. Three independent populations of 2 × 10^5^ HL60 RNAi p53 reporter cells were infected with virus at a transduction efficiency below 10% to ensure favor single retroviral integrations per cell, with approximately 200 independent integrations per shRNA contstruct. After 48 h of Puromycin selection and another 2 days of culture, mCherry positive (as a marker for library infected cells) cells displaying reduced GFP levels were sorted using a FACSAria cell sorter. One hundred cells per well were sorted directly into a 96-well PCR microplate (FS-C, Axygen Bioscienes) containing 10 μl 1× Thermopol buffer/proteinase K (100 μg/ml) (Sigma-Aldrich) per well. After sorting, the PCR plate was incubated for 30 min at 37°C, and then the enzyme was inactivated at 95°C for 10 min. Each well was added 10 μl 2× PCR mix (see below) to a final volume of 20 μl.

### Amplification of shRNA pools from sorted cells

#### PCR mixture

The PCR mixture for amplifying vector genomic DNA in a total volume of 20 μl contained 1.3 U and 0.1 U of Taq/Pfu DNA Polymerase (Fermentas), 1× Thermopol buffer (containing 20 mM of Tris–HCl (pH 8.8), 10 mM of KCl, 10 mM of (NH4) 2SO4, 2 mM of MgSO4, 0.1% of Triton X-100) (NEB), 0.25 μM of each primer, 0.375 mM dNTP mix (Fermentas). The reaction mixture for the second amplification round was the same as for the first one, except that 0.5 μM of the “inner” primers with tags were used instead of the “outer” primers. In the second amplification 2 μl of the first round PCR product was used and the reaction was also supplemented with 2.5 mM MgCl_2_. See Table 1 for primer information.

**Table 1:** Primers used in the amplification of shRNA pools from sorted cell

**Primer sequence**	**Id**	**Info**
CCGAGGTGGGCAGTCAATCAATCT	DK633outerr	outer primer
ACGCCATTTTGCAAGGCAT	DK637altouterf	outer primer
**454-adaptor primers**		
Lowercase letters provide a multiplexing code to allow separate PCR reactions to be pooled and sequenced together		
GCCTCCCTCGCGCCATCAGagcTTTCTTGGGTAGTTTGCAGTTT	DK621ACodeAGC	‘A’ type, nested
GCCTCCCTCGCGCCATCAGtgaTTTCTTGGGTAGTTTGCAGTTT	DK621ACodeTGA	‘A’ type, nested
GCCTCCCTCGCGCCATCAGcagTTTCTTGGGTAGTTTGCAGTTT	DK621ACodeCAG	‘A’ type, nested
Type A primers are identical except for the 3 nt’s in small letters		
GCCTTGCCAGCCCGCTCAGtactgacTTGTACAAGAAAGCTGGGTAAG	DK622BCodeTActgaC	‘B’ type, nested
GCCTTGCCAGCCCGCTCAGtacgatcTTGTACAAGAAAGCTGGGTAAG	DK622BCodeTAcgatC	‘B’ type, nested
GCCTTGCCAGCCCGCTCAGtacagtcTTGTACAAGAAAGCTGGGTAAG	DK622BCodeTAcagtC	‘B’ type, nested
GCCTTGCCAGCCCGCTCAGtagactcTTGTACAAGAAAGCTGGGTAAG	DK622BCodeTAgactC	‘B’ type, nested
GCCTTGCCAGCCCGCTCAGtagcatcTTGTACAAGAAAGCTGGGTAAG	DK622BCodeTAgcatC	‘B’ type, nested
GCCTTGCCAGCCCGCTCAGtacgtacTTGTACAAGAAAGCTGGGTAAG	DK622BCodeTAcgtaC	‘B’ type, nested
GCCTTGCCAGCCCGCTCAGtagctacTTGTACAAGAAAGCTGGGTAAG	DK622BCodeTAgctaC	‘B’ type, nested
GCCTTGCCAGCCCGCTCAGtagtcacTTGTACAAGAAAGCTGGGTAAG	DK622BCodeTAgtcaC	‘B’ type, nested
GCCTTGCCAGCCCGCTCAGcactgagTTGTACAAGAAAGCTGGGTAAG	DK622BCodeCActgaG	‘B’ type, nested
GCCTTGCCAGCCCGCTCAGcacgatgTTGTACAAGAAAGCTGGGTAAG	DK622BCodeCAcgatG	‘B’ type, nested
GCCTTGCCAGCCCGCTCAGcacagtgTTGTACAAGAAAGCTGGGTAAG	DK622BCodeCAcagtG	‘B’ type, nested
GCCTTGCCAGCCCGCTCAGcagactgTTGTACAAGAAAGCTGGGTAAG	DK622BCodeCAgactG	‘B’ type, nested
GCCTTGCCAGCCCGCTCAGcagcatgTTGTACAAGAAAGCTGGGTAAG	DK622BCodeCAgcatG	‘B’ type, nested
GCCTTGCCAGCCCGCTCAGcacgtagTTGTACAAGAAAGCTGGGTAAG	DK622BCodeCAcgtaG	‘B’ type, nested
GCCTTGCCAGCCCGCTCAGcagatcgTTGTACAAGAAAGCTGGGTAAG	DK622BCodeCAgatcG	‘B’ type, nested
GCCTTGCCAGCCCGCTCAGcagtcagTTGTACAAGAAAGCTGGGTAAG	DK622BCodeCAgtcaG	‘B’ type, nested
Type B primers are identical except the 7 nt’s in small letters.		

### Nested PCR

PCR thermal cycles were carried out using a thermal cycler (PTC-100, MJ Research) to amplify vector genomic DNA using the following sequences: preheat at 95°C for 5 min; 35 cycles of 95°C for 30 s, 58°C for 20 s and 72°C for 40 s. After 35 cycles of first round PCR, the second round PCR thermal cycles were performed: preheat at 95°C for 2 min; 20 cycles of 95°C for 20 s, 56°C for 20 s and 72°C for 30 s; additional extension at 72°C for 5 min to end the amplification for both programs. DNA was analyzed by 2% agarose gel electrophoresis and stained by ethidium bromide. 9 μl of each nested PCR were pooled, run on agarose gel and the PCR band (ca. 280 nt) was purified with Illustria GFX PCR DNA and gel band purification kit (GE Healthcare) before 454 sequencing (Roche) at The Norwegian High-Throughput Sequencing Centre (University of Oslo).

### 454 sequence data analysis

Raw FASTA format 454 sequence data were analysed using a series of simple Perl scripts (D. Micklem, unpublished). Initially, the sequences were scanned to determine orientation (sequencing off A or B-primer) and to identify the multiplex tag associated with each primer. Primer and constant vector sequences were then deleted and empty/too short sequences excluded. Remaining sequences were tested to determine whether they encoded a hairpin structure. Finally, the sequences were clustered into groups of near-identical sequences and the number of times each sequence was seen in each tag-group was tabulated.

### Immunoblotting

Cells were lysed in RIPA buffer (25 mM Tris-HCl pH 7.6, 150 mM NaCl, 1% NP-40, 1% sodium deoxycholate, 0.1% SDS) supplemented with protease inhibitor (13457200; Roche). After addition of lysis buffer, the extract was incubated for 5 min on ice, and then passed through a 29 gauge syringe. The suspension was centrifuged for 10 min at 10000 rpm and the supernatant protein concentration was measured using a BCA protein assay kit (#23227, Pierce). SDS/PAGE (Novex NuPAGE Bis-Tris 4-12% gels and MES buffer) and protein transfer were carried out according to standard procedures given by the manufacturer (Life Technologies). 20 μg protein was loaded on the gel. Western blots were developed with ECL substrate (#32106, Pierce) and the signal was detected and analyzed using the BioRad ChemiDoc XRS + system. p53 was detected using mouse monoclonal anti-human p53 antibody (ab1101, Abcam) and Glyceraldehyde 3-phosphate dehydrogenase (GAPDH) loading control was detected with Millipore MAB379 antibody (clone 6C5). Secondary antibody Goat anti-mouse (GAM) IgG HRP (#G21040, Invitrogen).

Intensity of individual bands was quantified using ImageJ densitometry software, andexpressed relative to GAPDH signal, as a measure of protein relative abundance in the different samples.

### Irradiation

L108 Luc, shp53-3, shp53-10 and shp53-13 HUVEC cells were harvested by trypsination and replated in 12- or 24-well plates 24 h before irradiation. The cells were irradiated on the plate with 2 or 8 Gy using a Philips RT 100 X-ray machine with an acceleration voltage of 100 kV.

### mRNA extraction

Irradiated cells were lysed by addition of 360 μl Buffer MRL (Qiagen)/well directly to the 24-well plates. mRNA was extracted and purified with a MagAttract Direct mRNA M48 Kit with oligo(dT) covered magnetic beads on a GenoM-48 Robotic Workstation (Qiagen). Standard settings for mRNA extraction were used with 100 μl elution volumes.

### cDNA synthesis

10 μl of mRNA was used as template for cDNA synthesis in a 20 μl reaction. The reaction buffer consisted of First Strand Buffer ×1, 10 mM dithiothretiol (DTT) and 5 U/μl SuperScriptTM II Reverse Transcriptase (Invitrogen) and 1 U/μl Protector RNase Inhibitor, 20 pmole/μl Hexanucleotide Mix and 0.25 mM of each dNTP, Li-Salt (Roche Diagnostics). The RT reaction was performed on a PTC-200 Peltier Thermal Cycler (MJ Research) at 22°C for 10 min, 42°C for 45 min and 99°C for 3 min.

### Quantitative PCR

TaqMan® Gene Expression Assays for p53, p21 and POLR2a (Pol II) were purchased from Applied Biosystems (Table 2). All primer products span exon–exon junctions to avoid amplification of genomic DNA. QPCR reactions were performed using 5 μl of cDNA in MicroAmp™ 96-well optical microtiter plates on a 7900HT Fast QPCR System in TaqMan® Fast Universal PCR Master Mix (Applied Biosystems) according to protocol (total volume 25 μl). cDNA was diluted 10 times prior to qPCR and all samples were run in duplicate. PCR results were analyzed with SDS 2.3 software (Applied Biosystems) and relative quantity was determined using the ΔΔCT Method [10], with DMSO-treated cells as calibrator and POLR2A as endogenous reference.

**Table 2:** Gene expression assays used for quantitative PCR

**Gene**	**Exon-exon boundary**	**Cat. number**
Cyclin-dependent kinase inhibitor 1A CDKN1A/P21	2-3	Hs00355782_m1
Tumor protein p53 TP53/p53	9-10	Hs00153349_m1
Polymerase (RNA) II (DNA directed) polypeptide A POLR2A	1-2	Hs00172187_m1

All primers/probes were purchased from Applied Biosystems.

### Gamma-H2AX staining

Gamma-H2AX detection was performed essentially as previously described [11]. H2AX is a form of Histone 2A that is phosphorylated upon DNA double strand break induction (gamma-H2AX) and can thus be used as a measure of DNA double strand breaks after irradiation. HUVEC cells were seeded in 12-well plates 24 h pre-irradiation. In brief, after irradiation the cells were incubated at 37°C for 30 min to allow complete H2AX phosphorylation and then kept on ice during the whole procedure to avoid phosphatase activity. After trypsination and harvest, the cells were incubated with Block-9 staining buffer containing anti-phospho-H2AX FITC-conjugated antibody (Millipore) and Vybrant Dye Cycle Violet stain (Invitrogen, Molecular Probes) for 3 h on ice. The increase in phosphorylated H2AX was then detected on a FACSAria flow cytometer (BD Biosciences). Excitation wavelengths were 488 nm/405 nm respectively and emission was detected with filter/bandpass 450/40 for FITC and 530/30 for Vybrant Dye Cycle Violet Stain.

### EdU staining

HUVEC cells were seeded out in 24-well plates 24 h pre-radiation. After irradiation, the cells were harvested by trypsination, diluted 1:8 and reseeded into 6-well plates. After growing for 48 h, EdU was added to the cells at a concentration of 10 μM. EdU assay, as a measure of cellular proliferation, was performed essentially as earlier described (Ref: Flow cytometry methods for DNA-damage induced clonogenic cell death calibrated to the colony forming assay, Yue Gao, Pegah Johansson, Aida Muslimovic and Ola Hammarsten, submitted manuscript), using a Click-iT® EdU Alexa Fluor® 488 Imaging Kit (Invitrogen). In brief, after 16 h incubation with EdU, the cells were washed with PBS and harvested by trypsination. After a 0.1% BSA/PBS wash, the cells were fixed for 15 min. The cells were washed again in BSA/PBS and then permeabilized for 30 min. After another wash with BSA/PBS, the cells were incubated in staining buffer according to protocol for 30 min, kept from light. After a final wash in 1× component E, the cells were incubated with Ribonuclease A for 30 min. Before Analysis on a FACSAria flow cytometer (BD Biosciences), the cells were diluted in 300 μl BSA/PBS.

### Data analysis

Statistical analysis was carried out using ANOVA followed by Tukey’s post hoc tests (GraphPad 4.0 Software, San Diego, CA). Significance was assumed for p value less than 0.05. Sigmoidal best fit curve generated with sigmoidal fit equation at http://zunzun.com, using the Hill with offset equation: ax^b/(c^b + x^b) + Offset.

## Results and discussion

In an effort to derive RNAi-competent sequences from a target gene we prepared an shRNA-expressing retroviral library based on random 21-26 nt sequences (~250 sequences) derived from the human Axl gene sequence, a receptor tyrosine kinase frequently expressed in human cancer and correlated with poor overall survival [12]. The shRNA library was transduced into MDA-MB-231 breast carcinoma cells; individual cells with reduced Axl cell surface expression were isolated by FACS and the cognate shRNA sequences identified by PCR and deep sequencing. We noted that each sequence displayed a unique, reproducible gene knockdown level that was not predictable by analysis in available RNAi sequence algorithms (Additional file 3: Figure S3). Collectively, these Axl-targeting shRNAs comprised an epi-allelic hypomorphic series in MDA-MB-231 cells that validated the role of Axl in metastasis *in vitro* and *in vivo*[2].

Based on these results we developed an approach to rapidly identify functional shRNA sequences from high-coverage gene specific shRNA libraries to enable epi-allelic hypomorphic analysis of putative therapeutic targets [1]. The natural fluctuation in protein levels routinely displayed by flow cytometric analysis can be modeled by normal probability distributions [13]. Hence, a cell population expressing an shRNA library against a target gene can be represented as a continuous distribution comprising multiple samples. The probability that a cell will be found below a given threshold is proportional to the difference in target gene expression means of the hypomorphic and wild type cell populations (Additional file 4: Figure S4). Importantly, this leads to the prediction that the number of cells expressing a specific shRNA found below this threshold will be related to the potency of the shRNA. This implies that the frequency of different shRNA sequences recovered from a sorted cell population should be proportional to their knockdown levels.

**Figure 1:**
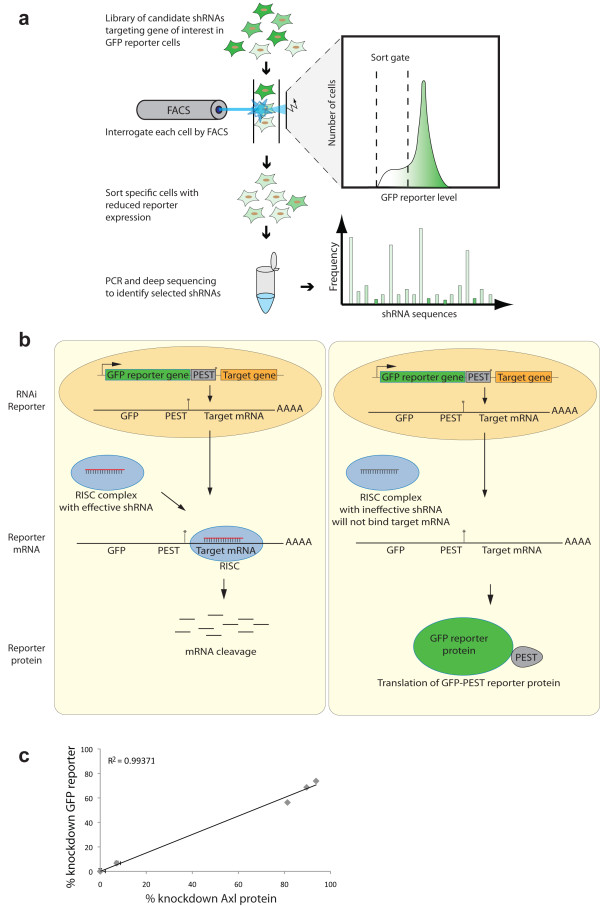
**The CellSelectRNAi approach. (a)** CellSelectRNAi principle. HL60 reporter cells are transduced with a library of candidate shRNAs targeting gene of interest. Each cell is interrogated by flow cytometry; cells with reduced reporter levels are sorted for subsequent PCR and shRNA sequence identification by 454 deep sequencing. Sequences are plotted by frequency representation in the sorted population. **(b)** Schematic of the CellSelectRNAi system. The RNAi reporter comprises a destabilized GFP (dsGFP), with the untranslated target gene cloned downstream of a translational stop codon (*). In the scenario on the left, a RISC complex is loaded with an effective shRNA that will bind to the target mRNA sequence in the reporter mRNA and initiate mRNA cleavage and prevention of dsGFP reporter translation. In the scenario on the right, the RISC complex is loaded with an ineffective shRNA that is unable to bind the target mRNA. The efficiency of each shRNA is proportional to the amount of dsGFP in the cell, and can be measured by flow cytometry. **(c)** RNAi reporter correlates with endogeneous gene expression. Flow cytometric analysis showing the level of RNAi reporter and endogeneous Axl cell surface protein in each epi-allelic MDA-MB-231 cell line demonstrates a linear relationship between RNAi reporter intensity and Axl protein stained with Axl antibody. Each point on the graph represents the geometric mean and the graph is representative of four individual experiments. The error bars represent 95% confidence-limit on the mean.

To test this prediction, we devised a FACS-based screening approach to identify functional RNAi-triggers (CellSelectRNAi; Figure 1a). Libraries of candidate retrovirally expressed shRNAs are transduced into a population of host cells stably expressing a dedicated RNAi-GFP reporter and sorted for target gene knockdown by FACS. shRNA sequences are identified from the sorted cells by PCR and deep sequencing, and assembled into a frequency profile. The frequency of a given shRNA should correlate with knockdown efficiency. The RNAi-GFP reporter, comprising an open reading frame encoding a destabilized GFP [14], fused to hygromycin phosphotransferase (hpt) together with the untranslated target cDNA sequence serving as a 3′-UTR may be used to identify functional shRNAs for any gene (Figure 1b). To validate this RNAi-reporter system we prepared an Axl RNAi-reporter MDA-MB-231 cell line that was transduced separately with four different Axl shRNAs (shAxl2, shAxl280, shAxl279, shAxl278) in addition to a control shRNA targeting luciferase (shLuc). These five cell lines were simultaneously analyzed for reporter and endogenous Axl surface receptor protein levels by multicolor flow cytometry. Axl RNAi-reporter GFP fluorescence correlated strongly (R^2^ = 0.9931) with endogeneous surface Axl receptor protein (Figure 1c) in the epi-allelic MDA-MB-231 cell series.

**Figure 2:**
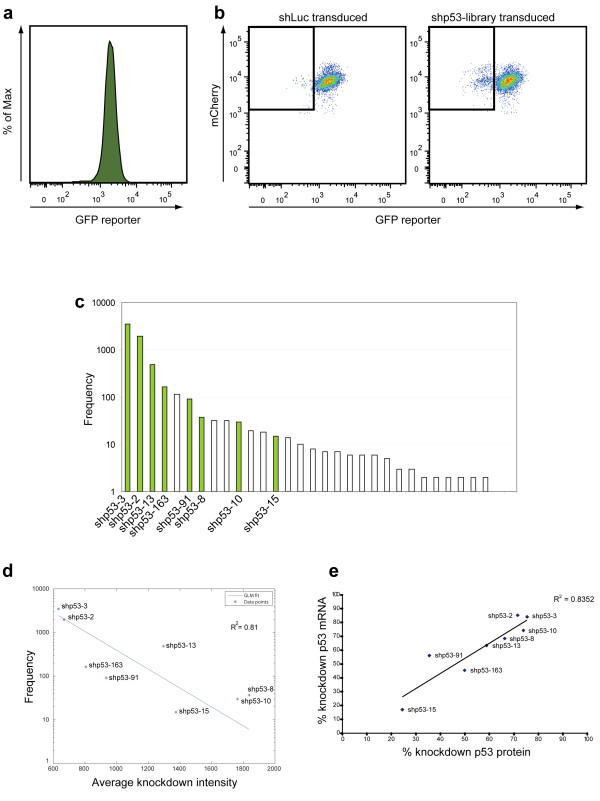
**Generation of an shp53 epi-allelic series. (a)** HL60 p53-RNAi reporter cell line. A HL60 cell line was transduced with p53-RNAi reporter construct and a single cell-derived clone was isolated by FACS, expanded and reanalysed for optimal (GFP) reporter expression by flow cytometry. **(b)** HL60 p53-RNAi reporter cells transduced with shp53 library show a population with reduced RNAi reporter expression. HL60 p53-RNAi reporter cells were transduced with a library of candidate p53 shRNA or control shRNA (shLuc). All cells were monitored by FACS and cells with reporter levels within the GFP_low_/RFP + gate (*box*) were sorted. **(c)** Functionally selected shRNA sequence frequency profile. Sequences retrieved from the 454 deep sequencing were tested to determine whether they encoded a hairpin structure and clustered into groups of near-identical sequences, and plotted by frequency. **(d)** Generalized linear model (GLM) fit of shRNA sequence frequency versus RNAi reporter level reveals a statistical relationship. Representative shp53-sequences were recloned, transduced into HL60 p53-RNAi reporter cells and analyzed by flow cytometry. A generalized linear model (GLM) with a log link function (‘log(frequency) ~ 1 + knockdown’) and Poisson distribution was applied to model the shRNA frequency versus RNAi reporter fluorescence intensity, generating estimated coefficients listed in Table 2, Additional file 4: Figure S4. The GLM model shows an exponential relationship between the observed shRNA frequency and measured RNAi reporter at a significance level of α = 0.05 (p ≈ 0). The reporter intensity data represents the mean of three independent experiments. **(e)** HUVE cells transduced with different shp53 clones generates an epi-allelic series. Transduced HUVE cells expressing different shp53 shRNA sequences show graded p53 expression levels as measured by relative quantification of Western blot versus mRNA levels.

To evaluate the CellSelectRNAi approach, we generated p53 RNAi-reporter expressing HL60 cells. Flow cytometric analysis of the HL60/p53-RNAi reporter cell line displayed dsGFP fluorescence with a mean fluorescence intensity of 1721 and coefficient of variation (CV) of 36.8 (Figure 2a). A high coverage p53 shRNA library was prepared using a novel cloning approach where random sequences derived from the p53 mRNA sequence are converted into a form that encodes a hairpin structure and cloned into a U6 promoter cassette within a self-inactivating retroviral vector (Additional file 5: Figure S5). The p53 shRNA library (approximately 100 unique sequences) was transduced into the HL60/p53-RNAi reporter cell line and analyzed by FACS (Figure 2b). A low transduction rate (~10%) was used to favor single retroviral integrations per cell. A sorting gate was set at 2 standard deviations from the mean of the wild type population and HL60/p53-RNAi reporter cells carrying p53-targeting shRNAs (RFP^+^) within the sorting gate were isolated (Figure 2b). 1800 sorting events were collected and proviral PCR amplification was conducted to generate a ~280 bp product that was analyzed by massively parallel pyrosequencing. The resulting 14790 shRNA sequences, of which 32 were p53 shRNA (Additional file 6: Figure S6) were rank ordered by frequency to generate a distribution plot (Figure 2c). Eight p53 shRNA sequences representing high, intermediate and low frequency levels were recloned and reintroduced into the HL60/p53-RNAi reporter cell line and monitored for reporter knockdown by flow cytometry. As predicted by the continuous distribution model, the shRNA sequence frequency derived from the sorted population showed a clear statistical relationship with the reporter intensity (Figure 2d). The most prevalent shRNA sequences identified by the CellSelectRNAi approach were the most potent also when measured at endogenous protein level and mRNA level (Figure 2e, Additional file 7: Figure S7). These results demonstrate accurate prediction of effective shRNA sequences by the CellSelectRNAi approach. Importantly, the most potent p53 shRNAs identified in the library screen are not predicted by available RNAi sequence algorithms [15] (Additional file 3: Figure S3).

**Figure 3:**
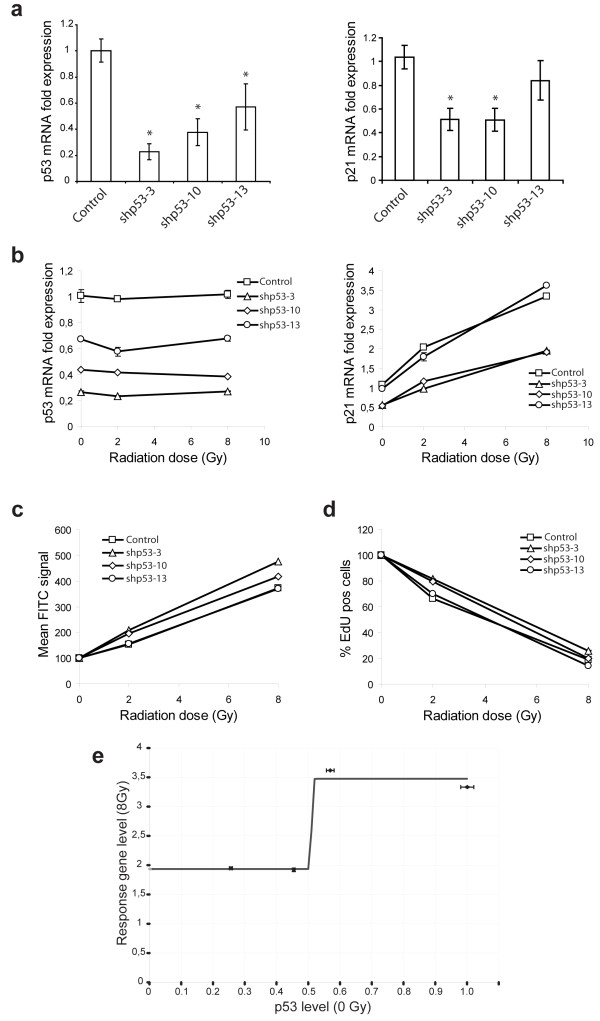
**Epi-allelic hypomorphic analysis to determine the threshold of p53-dependent responses to ionizing radiation. (a)** Non-linear correlation between p53 and p21 mRNA levels in p53 epi-allelic primary human endothelial cells. Graded p53 knockdown in human umbilical cord endothelial cells (HUVEC) with shp53-3, shp53-10 shp53-13 or negative control (shLuc) (*left panel*) corresponded to a non-linear reduction in p21 gene expression (*right panel*). Data represent the mean ± SD of three (p53) and five (p21) measurements representative for two independent experiments relative to control cells (set as 100%). Statistical analysis was carried out using ANOVA followed by Tukey’s post hoc tests, *P < 0,05. **(b)** Non-linear correlation between p53 and p21 mRNA levels in p53 epi-allelic primary human endothelial cells after irradiation. Irradiation of p53-epi-allelic HUVEC with 2 or 8 Gy did not affect p53 mRNA levels but increased p21 mRNA expression in a non-linear, p53-dependent manner. The data represents the mean of duplicate samples ± range. **(c)** Ionizing radiation induced H2AX phosphorylation in p53 epi-allelic primary human endothelial cells. H2AX phosphorylation, a measure of DNA double strand break induction, in HUVEC, correlates with greater p53 knockdown. The data represents the mean of duplicate samples ± range. **(d)** Cell proliferation in p53 epi-allelic primary human endothelial cells with shRNA against p53 after irradiation. The number of EdU positive p53-epi-allelic HUVEC following ionizing radiation, as a measure of cell division, increases with p53 knockdown efficiency. The data represents the mean of duplicate samples ± range. **(e)** Dose response relationship between p53-levels and radiation-induced p21 activation by epi-allelic analysis. Ionizing radiation-induced p21 activation displays a sigmoidal dose-dependency on p53 gene expression.

We conducted epi-allelic hypomorphic analysis using selected p53-targeting shRNAs to determine the threshold of p53-dependent responses to ionizing radiation in primary human endothelial cells. p53 is a transcription factor involved in the DNA damage response. p53 activation by DNA damage, such as upon ionizing radiation, results in increased expression of several inducible response genes. p21 is an example of a p53-regulated gene which is induced following ionizing radiation and was used here to assess the effects of shRNA-mediated knockdown on p53 activity [16]. As shown in Figure 3a-b, graded p53 knockdown in primary human endothelial cells resulted in a non-linear attenuation of p21-activation following ionizing radiation. Radiation-induced double-strand break repair (histone H2AX phosphorylation) and DNA synthesis were similarly diminished in endothelial cells with less than 50% of wild type p53 expression levels (Figure 3c-d). Intriguingly, the observed dose-dependency of ionizing radiation-induced p21-activation on p53 gene expression levels fits a sigmoidal dose response model with an apparent IC50 around 50% p53-expression, the expected gene dosage of a p53^+/-^ heterozygote (Figure 3e).

## Conclusion

These results demonstrate that flow cytometric selection of RNAi knockdown cells combined with deep sequence analysis can be used to identify multiple shRNA sequences that reduce target gene expression to predetermined levels. Perhaps surprisingly, the most potent functionally-selected sequences do not follow widely used RNAi scoring algorithms and are therefore not represented in current RNAi collections. The ability to readily generate several shRNAs per gene carries significant advantages. In addition to obviating the influence of “off-target” effects [17,18], a set of shRNAs with differing silencing potentials provides an epi-allelic series of hypomorphs, a genetic dose-response, that can uniquely relate gene expression to functional response thresholds and improve target-phenotype correlations [1,2]. Epi-allelic analysis highlights the “robustness” that buffers perturbations on biological systems and delineates drug target inhibition profiles for therapeutic translation [19].

## Abbreviations

dsGFP: Destabilized GFP; FACS: Fluorescence activated cell sorter; GFP: Green fluorescent protein; HUVEC: Human umbilical vein endothelial cell; LTR: Long terminal repeat; nt: Nucleotides; RFP: Red fluorescent protein; RNAi: RNA interference; shRNA: Short hairpin RNA.

## Competing interests

M.B. and D.R.M. are current employees of BerGenBio AS. J.B.L. is a founder, equity stockholder and consultant for BerGenBio AS. Aspects of the methodology presented are part of a patent (U. S. Patent No.: 8,735,064).

## Authors’ contributions

DRM, MB, OH and JBL designed the study; DRM and JBL developed the shRNA methodology; MB, PB, CT, TH and CG performed experiments; EH, PB and MB performed the statistical analysis; JBL, DRM and MB wrote the manuscript. All authors have read and approved the final manuscript.

## Supplementary Material

Additional file 1: Figure S1 Retroviral vector L101 complete sequence. Click here for file

Additional file 2: Figure S2 **(A)** L267 cloning vector with details of polylinker. Hairpins are expressed under the control of a modified U6 promoter, and cloned between the NaeI at the transcription start and the XcmI at the transcription terminator. PacI/HindIII restriction enzyme sites to facilitate cloning of the completed cassette into the final vector. **(B)** Structure of Hairpin Oligo DK540, including EcoP15I site (blue), GsuI sites (red) and a half-PvuII site (green). The loop sequence present in the completed hairpin cassette is in yellow. **(C)** Hairpin Oligo DK540 ligated to each end of a short fragment of p53. Digestion with EcoP13I releases two hairpin-tagged sequences which are then blunt-ended with Klenow fragment and dephosphorylated. **(D)** NaeI/XcmI-digested vector is ligated to haripin-tagged sequences. Because the haripin tag lacks a 5′ phosphate, the ligation product will have a ‘nick’ in the DNA phosphate backbone as indicated. DNA polymerisation from this nick using a strand-displacing polymerase opens out the hairpin into dsDNA. **(E) **Subsequent digestion with GsuI and recircularisation shortens the hairpin loop. Click here for file

Additional file 3: Figure S3 Functionally selected shRNA sequences fail in RNAi scoring algorithms. Hairpin sequences from this study that gave good knockdown efficiency (actual efficiency) were scored with eight different RNAi scoring algorithms using the i-Score server (http://www.med.nagoya-u.ac.jp/neurogenetics/i_Score/i_score.html). *Bold italic*: score within top 100 for gene. Glt: guide strand-loop-template strand orientation. Tlg: template strand-loop-guide strand orientation. Click here for file

Additional file 4: Figure S4 Predictions of knock-out effect. Click here for file

Additional file 5: Figure S5 Schematic drawing of the hairpin expression cassette. shRNAs are produced from the U6/tethracyclin-inducible (TetO) promoter. The U6 promoter can transcribe short RNAs by RNA Polymerase III. This expression cassette also contains genes coding for mCherry and Puromycine separated by a linker encoding the 2A self-cleaving sequence under the control of the EF1a promoter. Abbreviations: pA, polyadenylation signal; uro, puromycin; Ψ, packaging signal; T, terminator. Click here for file

Additional file 6: Figure S6 Sequences that have been scored as hairpins in screen. Lowercase letters signify hairpin loop. Click here for file

Additional file 7: Figure S7 Epiallelic HUVE cells comprising p53-targeting shRNA’s show graded p53 expression levels by immunoblotting. Click here for file
