# Supplementary material for: Flow cytometry-based functional selection of RNA interference triggers for efficient epi-allelic analysis of therapeutic targets
Source: BMC Biotechnol. 2014 Jun 21;14:57. doi: 10.1186/1472-6750-14-57 (PMC4074332; doi:10.1186/1472-6750-14-57)
Supplement: Additional file 1: Figure S1 — Retroviral vector L101 complete sequence. [file 1472-6750-14-57-S1.pdf]

Supplementary figure 1

```
LOCUS       L101                6902 bp ss-DNA     circular SYN 05-Sep-2004
DEFINITION  -
ACCESSION   -
KEYWORDS    -
SOURCE      -
FEATURES             Location/Qualifiers
     LTR             1..745
                     /note=CRU5
     promoter        1..589
                     /note=CMV immediate early promoter
     misc_feature    602..670
                     /note=5' R region
     misc_feature    671..745
                     /note=U5 region
     misc_signal     746..1656
                     /note=MoMLV extended packaging signal (psi+)
     misc_binding    747..764
                     /bound_moiety=tRNA-Pro
     misc_structure  805..829
                     /note=packaging stem-loop A
     misc_structure  879..910
                     /note=packaging stem-loop B
     misc_structure  911..953
                     /note=packaging stem-loop C
     misc_structure  956..975
                     /note=packaging stem-loop D
     gene            <1222..1647
                     /gene=gag
     CDS             <1222..1647
                     /gene=gag
                     /note=from Moloney murine leukemia virus
                     /codon_start=1
                     /product=Pr65 Gag polyprotein
                     /protein_id=ABN09913.1
                     /db_xref=GI:124365835

/translation=MGPGQTVTTPLSLTLGHWKDVERIAHNQSVDVKKRRWVTFCSAE

WPTFNVGWPRDGTFNRDLITQVKIKVFSPGPHGHPDQVPYIVTWEALAFDPPPWVKPF
                     VHPKPPPPLPPSAPSLPLEPPRSTPPRSSLYPALTPSLGA
     CDS             2060..4024
                     /note=GFP-PEST-2A-HygroR
     CDS             2909..2998
                     /note=FMDV 2A self-cleaving linker region
     CDS             2999..4024
                     /note=Hygromycin
     misc_feature    4025..4068
                     /note=MCS
     LTR             4116..4708
                     /note=3' LTR
     misc_feature    4116..4564
                     /note=U3 region
     misc_feature    4565..4633
                     /note=3' R region
     misc_feature    4634..4708
                     /note=U5 region
     rep_origin      5066..5778
                     /note=pMB1 ori (pUC)
     gene            complement(5843..6703)
                     /gene=bla
     CDS             complement(5843..6703)
                     /gene=bla
                     /function=ampicillin resistance
                     /codon_start=1
                     /product=beta-lactamase
                     /protein_id=ABN09912.1
                     /db_xref=GI:124365834
```

/translation=MSIQHFRVALIPFFAAFCLPVFAHPETLVKVKDAEDQLGARVGY

IELDLNSGKILESFRPEERFPMMSTFKVLLCGAVLSRIDAGQEQLGRRIHYSQNDLVE

YSPVTEKHLTDGMTVRELCSAAITMSDNTAANLLLTTIGGPKELTAFLHNMGDHVTRL

DRWEPELNEAIPNDERDTTMPVAMATTLRKLLTGELLTLASRQQLIDWMEADKVAGPL

LRSALPAGWFIADKSGAGERGSRGIIAALGPDGKPSRIVVIYTTGSQATMDERNRQIA
EIGASLIKHW

BASE COUNT 1589 A 1930 C 1784 G 1599 T 0 OTHER
ORIGIN ?

```
   1 CCTAGTTATT AATAGTAATC AATTACGGGG TCATTAGTTC ATAGCCCATA TATGGAGTTC
  61 CGCGTTACAT AACTTACGGT AAATGGCCCG CCTGGCTGAC CGCCCAACGA CCCCCGCCCA
 121 TTGACGTCAA TAATGACGTA TGTTCCCATA GTAACGCCAA TAGGGACTTT CCATTGACGT
 181 CAATGGGTGG AGTATTTACG GTAAACTGCC CACTTGGCAG TACATCAAGT GTATCATATG
 241 CCAAGTACGC CCCCTATTGA CGTCAATGAC GGTAAATGGC CCGCCTGGCA TTATGCCCAG
 301 TACATGACCT TATGGGACTT TCCTACTTGG CAGTACATCT ACGTATTAGT CATCGCTATT
 361 ACCATGGTGA TGCGGTTTTG GCAGTACATC AATGGGCGTG GATAGCGGTT TGACTCACGG
 421 GGATTTCCAA GTCTCCACCC CATTGACGTC AATGGGAGTT TGTTTTGGCA CCAAAATCAA
 481 CGGGACTTTC CAAAATGTCG TAACAACTCC GCCCCATTGA CGCAAATGGG CGGTAGGCAT
 541 GTACGGTGGG AGGTCTATAT AAGCAGAGCT CAATAAAAGA GCCCACAACC CCTCACTCGG
 601 CGCGCCAGTC CTCCGATTGA CTGAGTCGCC CGGGTACCCG TGTATCCAAT AAACCCTCTT
 661 GCAGTTGCAT CCGACTTGTG GTCTCGCTGT TCCTTGGGAG GGTCTCCTCT GAGTGATTGA
 721 CTACCCGTCA GCGGGGGTCT TTCATTTGGG GGCTCGTCCG GGATCGGGAG ACCCCTGCCC
 781 AGGGACCACC GACCCACCAC CGGGAGGTAA GCTGGCCAGC AACTTATCTG TGTCTGTCCG
 841 ATTGTCTAGT GTCTATGACT GATTTTATGC GCCTGCGTCG GTACTAGTTA GCTAACTAGC
 901 TCTGTATCTG GCGGACCCGT GGTGGAACTG ACGAGTTCGG AACACCCGGC CGCAACCCTG
 961 GGAGACGTCC CAGGGACTTC GGGGGCCGTT TTTGTGGCCC GACCTGAGTC CAAAAATCCC
1021 GATCGTTTTG GACTCTTTGG TGCACCCCCC TTAGAGGAGG GATATGTGGT TCTGGTAGGA
1081 GACGAGAACC TAAAACAGTT CCCGCCTCCG TCTGAATTTT TGCTTTCGGT TTGGGACCGA
1141 AGCCGCGCCG CGCGTCTTGT CTGCTGCAGC ATCGTTCTGT GTTGTCTCTG TCTGACTGTG
1201 TTTCTGTATT TGTCTGAAAA TATGGGCCCG GGCCAGACTG TTACCACTCC CTTAAGTTTG
1261 ACCTTAGGTC ACTGGAAAGA TGTCGAGCGG ATCGCTCACA ACCAGTCGGT AGATGTCAAG
1321 AAGAGACGTT GGGTTACCTT CTGCTCTGCA GAATGGCCAA CCTTTAACGT CGGATGGCCG
1381 CGAGACGGCA CCTTTAACCG AGACCTCATC ACCCAGGTTA AGATCAAGGT CTTTTCACCT
1441 GGCCCGCATG GACACCCAGA CCAGGTCCCC TACATCGTGA CCTGGGAAGC CTTGGCTTTT
1501 GACCCCCCTC CCTGGGTCAA GCCCTTTGTA CACCCTAAGC CTCCGCCTCC TCTTCCTCCA
1561 TCCGCCCCGT CTCTCCCCCT TGAACCTCCT CGTTCGACCC CGCCTCGATC CTCCCTTTAT
1621 CCAGCCCTCA CTCCTTCTCT AGGCGCCCCC ATATGGCCAT ATGAGATCGA TCTTATATGG
1681 GGCACCCCCG CCCCTTGTAA ACTTCCCTGA CCCTGACATG ACAAGAGTTA CTAACAGCCC
1741 CTCTCTCCAA GCTCACTTAC AGGCTCTCTA CTTAGTCCAG CACGAAGTCT GGAGACCTCT
1801 GGCGGCACGT ACCCAAGAAC AACTGGACCG ACCGGTGGTA CCTCACCCTT ACCGAGTCGG
1861 CGACACAGTG TGGGTCCGCC GACACCAGAC TAAGAACCTA GAACCTCGCT GGAAAGGACC
1921 TTACACAGTC CTGCTGACCA CCCCCACCGC CCTCAAAGTA GACGGCATCG CAGCTTGGAT
1981 ACACGCCGCC CACGTGAAGG CTGCCGACCC CGGGGGTGGA CCATCCTCTA GACTGCCGGA
2041 TCTCGAGGGA TCCACCACCA TGGTGAGCAA GGGCGAGGAG CTGTTCACCG GGGTGGTGCC
2101 CATCCTGGTC GAGCTGGACG GCGACGTAAA CGGCCACAAG TTCAGCGTGT CCGGCGAGGG
2161 CGAGGGCGAT GCCACCTACG GCAAGCTGAC CCTGAAGTTC ATCTGCACCA CCGGCAAGCT
2221 GCCCGTGCCC TGGCCCACCC TCGTGACCAC CCTGACCTAC GGCGTGCAGT GCTTCAGCCG
2281 CTACCCCGAC CACATGAAGC AGCACGACTT CTTCAAGTCC GCCATGCCCG AAGGCTACGT
2341 CCAGGAGCGC ACCATCTTCT TCAAGGACGA CGGCAACTAC AAGACCCGCG CCGAGGTGAA
2401 GTTCGAGGGC GACACCCTGG TGAACCGCAT CGAGCTGAAG GGCATCGACT TCAAGGAGGA
2461 CGGCAACATC CTGGGGCACA AGCTGGAGTA CAACTACAAC AGCCACAACG TCTATATCAT
2521 GGCCGACAAG CAGAAGAACG GCATCAAGGT GAACTTCAAG ATCCGCCACA ACATCGAGGA
2581 CGGCAGCGTG CAGCTCGCCG ACCACTACCA GCAGAACACC CCCATCGGCG ACGGCCCCGT
2641 GCTGCTGCCC GACAACCACT ACCTGAGCAC CCAGTCCGCC CTGAGCAAAG ACCCCAACGA
2701 GAAGCGCGAT CACATGGTCC TGCTGGAGTT CGTGACCGCC GCCGGGATCA CTCTCGGCAT
2761 GGACGAGCTG TACAAGGAAT TGAGTCATGG CTTCCCGCCG GAGGTGGAGG AGCAGGATGA
2821 TGGCACGCTG CCCATGTCTT GTGCCCAGGA GAGCGGGATG GACCGTCACC CTGCAGCCTG
2881 TGCTTCTGCT AGGATCAATG TGGAATTCGG AGGTGGCAGC GGTGGCGGTC AGCTGTTGAA
2941 TTTTGACCTT CTTAAACTTG CGGGAGACGT CGAGTCCAAC CCTGGGCCCA CCACCACCAT
3001 GAAAAAGCCT GAACTCACCG CGACGTCTGT CGAGAAGTTT CTGATCGAAA AGTTCGACAG
3061 CGTCTCCGAC CTGATGCAGC TCTCGGAGGG CGAAGAATCT CGTGCTTTCA GCTTCGATGT
3121 AGGAGGGCGT GGATATGTCC TGCGGGTAAA TAGCTGCGCC GATGGTTTCT ACAAAGATCG
3181 TTATGTTTAT CGGCACTTTG CATCGGCCGC GCTCCCGATT CCGGAAGTGC TTGACATTGG
3241 GGAATTTAGC GAGAGCCTGA CCTATTGCAT CTCCCGCCGT GCACAGGGTG TCACGTTGCA
3301 AGACCTGCCT GAAACCGAAC TGCCCGCTGT TCTGCAGCCG GTCGCGGAGG CCATGGATGC
3361 GATCGCTGCG GCCGATCTTA GCCAGACGAG CGGGTTCGGC CCATTCGGAC CGCAAGGAAT
```

3421 CGGTCAATAC ACTACATGGC GTGATTTCAT ATGCGCGATT GCTGATCCCC ATGTGTATCA
3481 CTGGCAAACT GTGATGGACG ACACCGTCAG TGCGTCCGTC GCGCAGGCTC TCGATGAGCT
3541 GATGCTTTGG GCCGAGGACT GCCCCGAAGT CCGGCACCTC GTGCACGCGG ATTTCGGCTC
3601 CAACAATGTC CTGACGGACA ATGGCCGCAT AACAGCGGTC ATTGACTGGA GCGAGGCGAT
3661 GTTCGGGGAT TCCCAATACG AGGTCGCCAA CATCTTCTTC TGGAGGCCGT GGTTGGCTTG
3721 TATGGAGCAG CAGACGCGCT ACTTCGAGCG GAGGCATCCG GAGCTTGCAG GATCGCCGCG
3781 GCTCCGGGCG TATATGCTCC GCATTGGTCT TGACCAACTC TATCAGAGCT TGGTTGACGG
3841 CAATTTCGAT GATGCAGCTT GGGCGCAGGG TCGATGCGAC GCAATCGTCC GATCCGGAGC
3901 CGGGACTGTC GGGCGTACAC AAATCGCCCG CAGAAGCGCG GCCGTCTGGA CCGATGGCTG
3961 TGTAGAAGTA CTCGCCGATA GTGGAAACCG ACGCCCCAGC ACTCGTCCGA GGGCAAAGGA
4021 ATAGTCGACA AGCTTCAATT GTTAACAGAT CTCCTAGGTA GCGGCCGCTC GACGATAAAA
4081 TAAAAGATTT TATTTAGTCT CCAGAAAAAG GGGGGAATGA AAGACCCCAC CTGTAGGTTT
4141 GGCAAGCTAG CTTAAGTAAC GCCATTTTGC AAGGCATGGA AAAATACATA ACTGAGAATA
4201 GAGAAGTTCA GATCAAGGTC AGGAACAGAT GGAACAGCTG AATATGGGCC AAACAGGATA
4261 TCTGTGGTAA GCAGTTCCTG CCCCGGCTCA GGGCCAAGAA CAGATGGAAC AGCTGAATAT
4321 GGGCCAAACA GGATATCTGT GGTAAGCAGT TCCTGCCCCG GCTCAGGGCC AAGAACAGAT
4381 GGTCCCCAGA TGCGGTCCAG CCCTCAGCAG TTTCTAGAGA ACCATCAGAT GTTTCCAGGG
4441 TGCCCCAAGG ACCTGAAATG ACCCTGTGCC TTATTTGAAC TAACCAATCA GTTCGCTTCT
4501 CGCTTCTGTT CGCGCGCTTC TGCTCCCCGA GCTCAATAAA AGAGCCCACA ACCCCTCACT
4561 CGGGGCGCCA GTCCTCCGAT TGACTGAGTC GCCCGGGTAC CCGTGTATCC AATAAACCCT
4621 CTTGCAGTTG CATCCGACTT GTGGTCTCGC TGTTCCTTGG GAGGGTCTCC TCTGAGTGAT
4681 TGACTACCCG TCAGCGGGGG TCTTTCATTT CCGACTTGTG GTCTCGCTGC CTTGGGAGGG
4741 TCTCCTCTGA GTGATTGACT ACCCGTCAGC GGGGGTCTTC ACATGCAGCA TGTATCAAAA
4801 TTAATTTGGT TTTTTTTCTT AAGTATTTAC ATTAAATGGC CATAGTTGCA TTAATGAATC
4861 GGCCAACGCG CGGGGAGAGG CGGTTTGCGT ATTGGCGCTC TTCCGCTTCC TCGCTCACTG
4921 ACTCGCTGCG CTCGGTCGTT CGGCTGCGGC GAGCGGTATC AGCTCACTCA AAGGCGGTAA
4981 TACGGTTATC CACAGAATCA GGGGATAACG CAGGAAAGAA CATGTGAGCA AAAGGCCAGC
5041 AAAAGGCCAG GAACCGTAAA AAGGCCGCGT TGCTGGCGTT TTTCCATAGG CTCCGCCCCC
5101 CTGACGAGCA TCACAAAAAT CGACGCTCAA GTCAGAGGTG GCGAAACCCG ACAGGACTAT
5161 AAAGATACCA GGCGTTTCCC CCTGGAAGCT CCCTCGTGCG CTCTCCTGTT CCGACCCTGC
5221 CGCTTACCGG ATACCTGTCC GCCTTTCTCC CTTCGGGAAG CGTGGCGCTT TCTCATAGCT
5281 CACGCTGTAG GTATCTCAGT TCGGTGTAGG TCGTTCGCTC CAAGCTGGGC TGTGTGCACG
5341 AACCCCCCGT TCAGCCCGAC CGCTGCGCCT TATCCGGTAA CTATCGTCTT GAGTCCAACC
5401 CGGTAAGACA CGACTTATCG CCACTGGCAG CAGCCACTGG TAACAGGATT AGCAGAGCGA
5461 GGTATGTAGG CGGTGCTACA GAGTTCTTGA AGTGGTGGCC TAACTACGGC TACACTAGAA
5521 GGACAGTATT TGGTATCTGC GCTCTGCTGA AGCCAGTTAC CTTCGGAAAA AGAGTTGGTA
5581 GCTCTTGATC CGGCAAACAA ACCACCGCTG GTAGCGGTGG TTTTTTTGTT TGCAAGCAGC
5641 AGATTACGCG CAGAAAAAAA GGATCTCAAG AAGATCCTTT GATCTTTTCT ACGGGGTCTG
5701 ACGCTCAGTG GAACGAAAAC TCACGTTAAG GGATTTTGGT CATGAGATTA TCAAAAAGGA
5761 TCTTCACCTA GATCCTTTTA AATTAAAAAT GAAGTTTGCG CAAATCAATC TAAAGTATAT
5821 ATGAGTAAAC TTGGTCTGAC AGTTACCAAT GCTTAATCAG TGAGGCACCT ATCTCAGCGA
5881 TCTGTCTATT TCGTTCATCC ATAGTTGCCT GACTCCCCGT CGTGTAGATA ACTACGATAC
5941 GGGAGGGCTT ACCATCTGGC CCCAGTGCTG CAATGATACC GCGAGACCCA CGCTCACCGG
6001 CTCCAGATTT ATCAGCAATA AACCAGCCAG CCGGAAGGGC CGAGCGCAGA AGTGGTCCTG
6061 CAACTTTATC CGCCTCCATC CAGTCTATTA ATTGTTGCCG GGAAGCTAGA GTAAGTAGTT
6121 CGCCAGTTAA TAGTTTGCGC AACGTTGTTG CCATTGCTAC AGGCATCGTG GTGTCACGCT
6181 CGTCGTTTGG TATGGCTTCA TTCAGCTCCG GTTCCCAACG ATCAAGGCGA GTTACATGAT
6241 CCCCCATGTT GTGCAAAAAA GCGGTTAGCT CCTTCGGTCC TCCGATCGTT GTCAGAAGTA
6301 AGTTGGCCGC AGTGTTATCA CTCATGGTTA TGGCAGCACT GCATAATTCT CTTACTGTCA
6361 TGCCATCCGT AAGATGCTTT TCTGTGACTG GTGAGTACTC AACCAAGTCA TTCTGAGAAT
6421 AGTGTATGCG GCGACCGAGT TGCTCTTGCC CGGCGTCAAT ACGGGATAAT ACCGCGCCAC
6481 ATAGCAGAAC TTTAAAAGTG CTCATCATTG GAAAACGTTC TTCGGGGCGA AAACTCTCAA
6541 GGATCTTACC GCTGTTGAGA TCCAGTTCGA TGTAACCCAC TCGTGCACCC AACTGATCTT
6601 CAGCATCTTT TACTTTCACC AGCGTTTCTG GGTGAGCAAA AACAGGAAGG CAAAATGCCG
6661 CAAAAAAGGG AATAAGGGCG ACACGGAAAT GTTGAATACT CATACTCTTC CTTTTTCAAT
6721 ATTATTGAAG CATTTATCAG GGTTATTGTC TCATGAGCGG ATACATATTT GAATGTATTT
6781 AGAAAAATAA ACAAATAGGG GTTCCGCGCA CATTTCCCCG AAAAGTGCCA CCAGCTTTGC
6841 TCTTAGGAGT TTCCTAATAC ATCCCAAACT CAAATATATA AAGCATTTGA CTTGTTCTAT
6901 GC
//
