# Supplementary material for: Flow cytometry-based functional selection of RNA interference triggers for efficient epi-allelic analysis of therapeutic targets
Source: BMC Biotechnol. 2014 Jun 21;14:57. doi: 10.1186/1472-6750-14-57 (PMC4074332; doi:10.1186/1472-6750-14-57)
Supplement: Additional file 2: Figure S2 — (A) L267 cloning vector with details of polylinker. Hairpins are expressed under the control of a modified U6 promoter, and cloned between the NaeI at the transcription start and the XcmI at the transcription terminator. PacI/HindIII restriction enzyme sites to facilitate cloning of the completed cassette into the final vector. (B) Structure of Hairpin Oligo DK540, including EcoP15I site (blue), GsuI sites (red) and a half-PvuII site (green). The loop sequence present in the completed hairpin cassette is in yellow. (C) Hairpin Oligo DK540 ligated to each end of a short fragment of p53. Digestion with EcoP13I releases two hairpin-tagged sequences which are then blunt-ended with Klenow fragment and dephosphorylated. (D) NaeI/XcmI-digested vector is ligated to haripin-tagged sequences. Because the haripin tag lacks a 5′ phosphate, the ligation product will have a ‘nick’ in the DNA phosphate backbone as indicated. DNA polymerisation from this nick using a strand-displacing polymerase opens out the hairpin into dsDNA. (E) Subsequent digestion with GsuI and recircularisation shortens the hairpin loop. [file 1472-6750-14-57-S2.pdf]

Supplementary figure 2

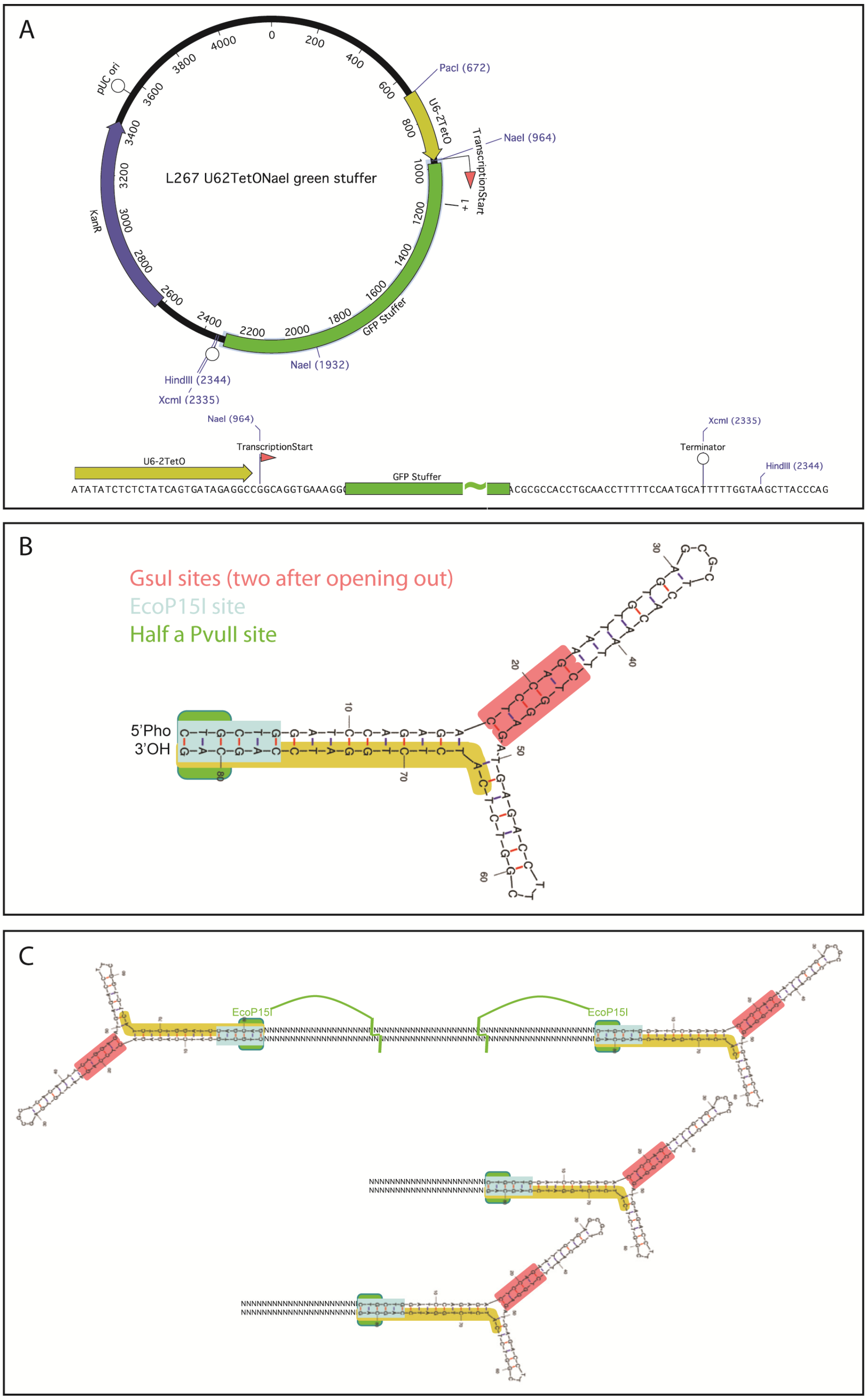

D

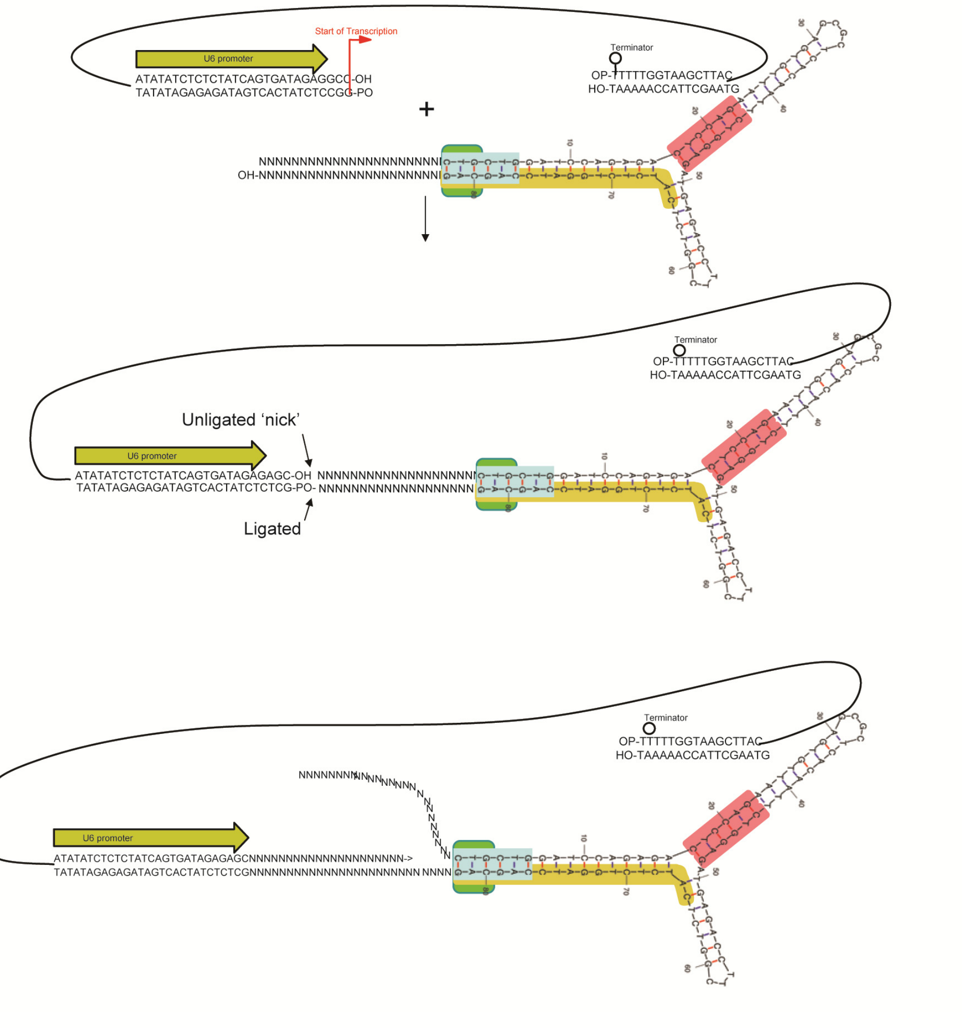
Start of Transcription
U6 promoter
ATATATCTCTCTATCAGTGATAGAGGCC-OH
TATATAGAGAGATAGTCACTATCTCCGG-PO
Terminator
OP-TTTTTGGTAAGCTTAC
HO-TAAAAACCATTCGAATG
+
Unligated 'nick'
ATATATCTCTCTATCAGTGATAGAGAGC-OH
TATATAGAGAGATAGTCACTATCTCTCG-PO-
Ligated

E

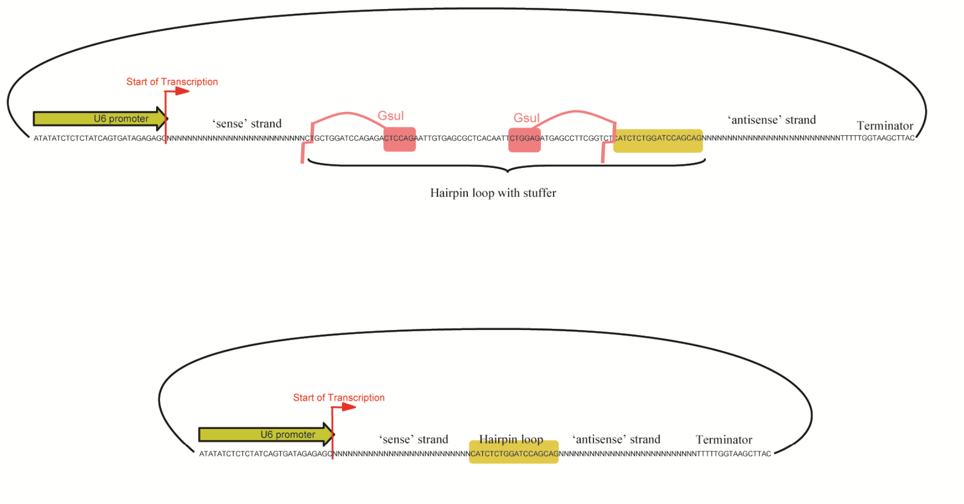
Start of Transcription
U6 promoter
'sense' strand
GsuI
GsuI
'antisense' strand
Terminator
Hairpin loop with stuffer
Start of Transcription
U6 promoter
'sense' strand
Hairpin loop
'antisense' strand
Terminator

.

(A) L267 cloning vector with details of polylinker. Hairpins are expressed under the control of a modified U6 promoter, and cloned between the NaeI at the transcription start and the XcmI at the transcription terminator. PacI/HindIII restriction enzyme sites to facilitate cloning of the completed cassette into the final vector. (B) Structure of Hairpin Oligo DK540, including EcoP15I site (blue), GsuI sites (red) and a half-PvuII site (green). The loop sequence present in the completed hairpin cassette is in yellow.
(C) Hairpin Oligo DK540 ligated to each end of a short fragment of p53. Digestion with EcoP13I releases two hairpin-tagged sequences which are then blunt-ended with Klenow fragment and dephosphorylated. (D) NaeI/XcmI-digested vector is ligated to haripin-tagged sequences. Because the haripin tag lacks a 5' phosphate, the ligation product will have a 'nick' in the DNA phosphate backbone as indicated. DNA polymerisation from this nick using a strand-displacing polymerase opens out the hairpin into dsDNA. (E) Subsequent digestion with GsuI and recircularisation shortens the hairpin loop.
