# Supplementary material for: Flow cytometry-based functional selection of RNA interference triggers for efficient epi-allelic analysis of therapeutic targets
Source: BMC Biotechnol. 2014 Jun 21;14:57. doi: 10.1186/1472-6750-14-57 (PMC4074332; doi:10.1186/1472-6750-14-57)
Supplement: Additional file 3: Figure S3 — Functionally selected shRNA sequences fail in RNAi scoring algorithms. Hairpin sequences from this study that gave good knockdown efficiency (actual efficiency) were scored with eight different RNAi scoring algorithms using the i-Score server (http://www.med.nagoya-u.ac.jp/neurogenetics/i_Score/i_score.html). Bold italic: score within top 100 for gene. Glt: guide strand-loop-template strand orientation. Tlg: template strand-loop-guide strand orientation. [file 1472-6750-14-57-S3.pdf]

shRNA ID	#hits in screen	Pos. cDNA	Sense		Ui-Tei	Amarz-guioui	Hsieh	Taka-saki	s-Biopredsi	i-Score	Rey-nolds	Katoh	DSIR	%KD
shp53-3	3462	520	CACATGTAGTTGTAGTGGATGGTGGT	glt	II	1	-1	0.3	0.676	53.2	4	60.5	75.6	>90
shp53-2	1959	369	GCGCTGCTCAGATAGCGATGGTCTGG	tlg	II	0	-1	6.1	0.36	41.2	0	48.4	66.2	>90
shp53-13	486	1365	CTGTTGAATTTTCTCTAACTTCAAGG	tlg	***Ia***	3	***2***	9.4	***0.812***	***65.3***	***7***	***83.2***	84.1	>70
Axl2	-	762	GACATCCTCTTTCTCCTGCGAAGCCCAT	tlg	II	1	2	5	0.729	60	6	44.3	79	>95
Axl278	-	1587	ACGGGTCTCCTTCTTTCGCCG	glt	II	2	0	3	0.524	42	0	59.9	66.1	>80
Axl280	-	221	ATGCACGCCCAGCCGCACAGCG	glt	II	1	1	2.7	0.3	32.6	-1	17.9	49.7	>95

Supplementary figure 3. Functionally selected shRNA sequences fail in RNAi scoring algorithms. Hairpin sequences from this study that gave good knockdown efficiency (actual efficiency) were scored with eight different RNAi scoring algorithms using the i-Score server (http://www.med.nagoya-u.ac.jp/neurogenetics/i_Score/i_score.html). ***Bold italic***: score within top 100 for gene. Glt: guide strand-loop-template strand orientation. Tlg: template strand-loop-guide strand orientation.
