# Supplementary material for: Flow cytometry-based functional selection of RNA interference triggers for efficient epi-allelic analysis of therapeutic targets
Source: BMC Biotechnol. 2014 Jun 21;14:57. doi: 10.1186/1472-6750-14-57 (PMC4074332; doi:10.1186/1472-6750-14-57)
Supplement: Additional file 4: Figure S4 — Predictions of knock-out effect. [file 1472-6750-14-57-S4.pdf]

Supplementary figure 4

Predictions of knock-out effect

Assume multiple normally distributed continuous sub-populations X_i around their means μ_i for all $i = \{1,\ldots,N\}$, where N is the number of sub-populations. The sub-populations together constitute the main population X with average μ.

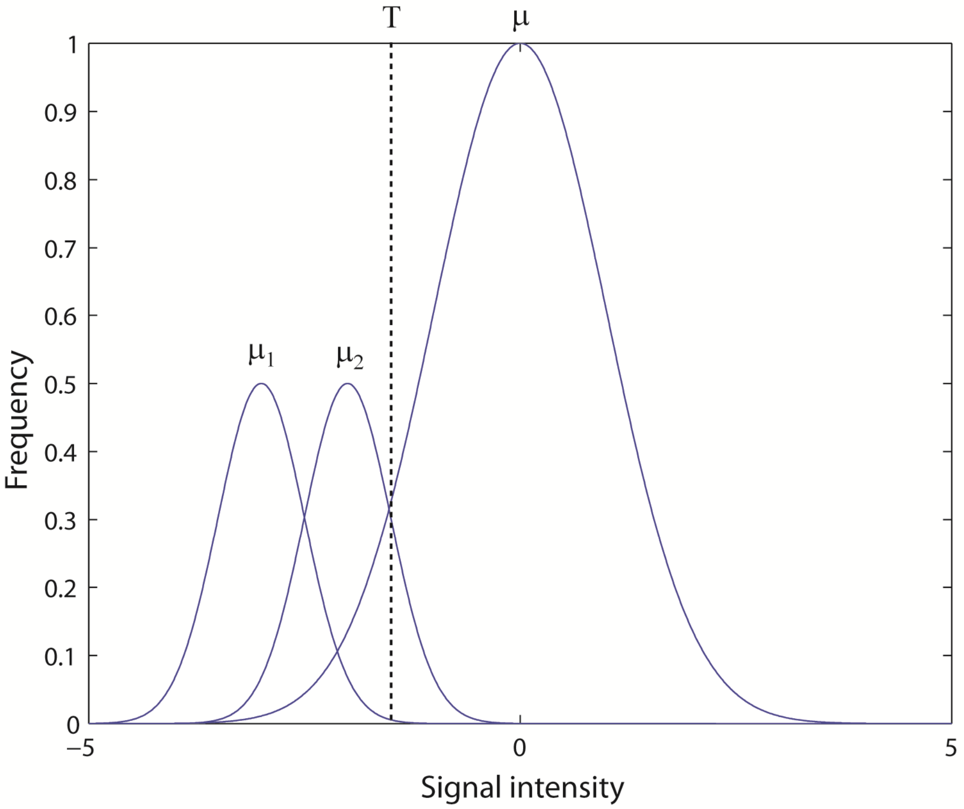

Figure 1 The distribution of a main populations and its subpopulations. The gating threshold is set at the dashed line.

Let $\mu_i < \mu_{i+1}$ be sorted in increasing order. Also, assume non-equal standard deviation for each subpopulation $\sigma_i \neq \sigma_j \forall i, j = \{1,\ldots,n\}$. Consider two neighbouring subpopulations X_1 and X_2. The probability of picking an element from subpopulation X_i or X_{i+1} respectively, is

$$P(-\infty < X_i < T) \equiv P_i = \frac{1}{\sqrt{2\pi}\sigma_i} \int_{-\infty}^{T} e^{-(x-\mu_i)^2/(2\sigma_i)} dx$$

$$P(-\infty < X_{i+1} < T) \equiv P_{i+1} = \frac{1}{\sqrt{2\pi}\sigma_{i+1}} \int_{-\infty}^{T} e^{-(x-\mu_{i+1})^2/(2\sigma_{i+1})} dx$$

Clearly $P_i > P_{i+1}$ for a continuous distribution, thus we collect more cells from sample X_i than from X_{i+1} when gating at T. Therefore, we expect to collect more cells from samples with lower means. However, from the main population we practically collect only a *finite* number of cells. Thus, we want to establish a relation between the sampled cells and the order in knockdown efficiency. Let n_i be the number of cells taken from each sub-population, and s_i

is the sample standard deviation. We want to investigate whether the order of means $\bar{X}_i < \bar{X}_{i+1} \forall i, j = \{1,\ldots,n\}$ may randomly change upon resampling within a 99% probability where $\bar{X}_i$ is the mean of a random sample taken from subpopulation X_i. Since $\bar{X}_i$ and $\bar{X}_{i+1}$ are normally distributed, the difference $\bar{X}_i - \bar{X}_{i+1}$ is still normally distributed. A fixed number of cells is picked from the main population, with a known number of cells n_i from each sub-population. Assuming that the sample standard deviations are unequal and unknown, the upper confidence bound for the difference between any two $\bar{X}_i$ and $\bar{X}_{i+1}$ requires

$$(\bar{X}_i - \bar{X}_{i+1}) + t_{\alpha/2}\sqrt{\frac{s_i^2}{n_i} + \frac{s_{i+1}^2}{n_{i+1}}} < 0$$

We also assume at least 120 degrees of freedom, which is fulfilled for our data. This confidence bound will ensure a $1-\alpha$ probability that $\bar{X}_i$ is lower than $\bar{X}_{i+1}$, and the order will therefore not be switched. Applying this approach to our knockdown data, we obtain confidence intervals between all eight trials as presented in Table 1.

Table 1. Upper confidence limits ($\alpha = 0.01$) for the difference between two average knockdown efficiencies. One would expect the upper confidence limit to become larger than zero if the knockdown efficiency order was to be switched around upon resampling. This is not fulfilled for any of comparisons, hence the order obtained is likely to be found in repeated experiments.

	3	2	163	91	13	15	10	8
3	-	-0.0251	-0.1613	-0.2873	-0.6385	-0.7161	-1.0884	-1.1588
2	-	-	-0.1263	-0.2523	-0.6035	-0.6811	-1.0533	-1.1237
163	-	-	-	-0.1125	-0.4642	-0.5419	-0.9145	-0.9849
91	-	-	-	-	-0.3344	-0.4121	-0.7852	-0.8557
13	-	-	-	-	-	-0.0442	-0.4193	-0.4901
15	-	-	-	-	-	-	-0.3393	-0.4101
10	-	-	-	-	-	-	-	-0.0114
8	-	-	-	-	-	-	-	-

According to Table 1 seems to be no strict order dependency of the knockdown with the frequency. However, there still may be a statistical relationship. Applying a generalized linear model (GLM) with a log link function ('log(frequency) ~ 1 + knockdown') gives the estimated coefficients as shown in Table 2.

Table 2. GLM fit frequencey versus knockdown. The GLM model demonstrates a clear relation between the frequency and the knockdown efficiency at a significance level of 0.05 ($p \approx 0$).

	Estimate	SE	t	p
(Intercept)	10.956	0.064349	170.26	0
Knockdown	-0.0049844	8.7816e-05	-56.76	0

This analysis clearly reveals a statistical relationship between the knockdown efficiency and the frequency. The GLM fit and the observed data points are shown in Figure 2.

[1] Ronald E. Walpole, Raymond H. Myers, Sharon L. Myers, Keying Ye. "Probability & Statistics for Engineers & Scientists." Seventh edition, 2002, Prentice-Hall.
