# Supplementary material for: Flow cytometry-based functional selection of RNA interference triggers for efficient epi-allelic analysis of therapeutic targets
Source: BMC Biotechnol. 2014 Jun 21;14:57. doi: 10.1186/1472-6750-14-57 (PMC4074332; doi:10.1186/1472-6750-14-57)
Supplement: Additional file 5: Figure S5 — Schematic drawing of the hairpin expression cassette. shRNAs are produced from the U6/tethracyclin-inducible (TetO) promoter. The U6 promoter can transcribe short RNAs by RNA Polymerase III. This expression cassette also contains genes coding for mCherry and Puromycine separated by a linker encoding the 2A self-cleaving sequence under the control of the EF1a promoter. Abbreviations: pA, polyadenylation signal; uro, puromycin; Ψ, packaging signal; T, terminator. [file 1472-6750-14-57-S5.pdf]

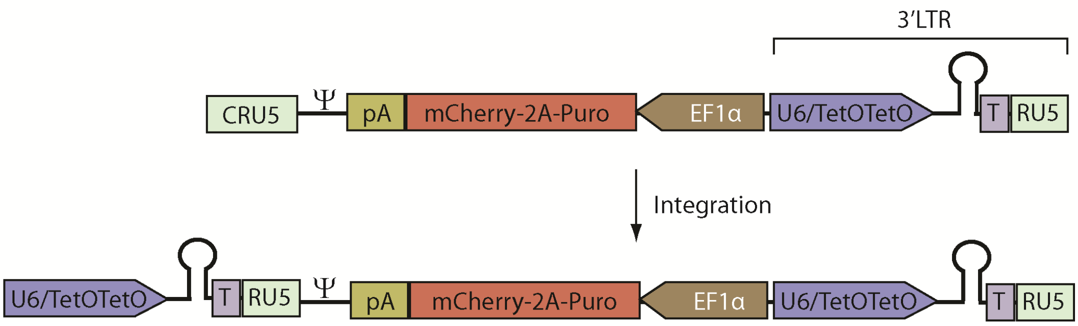

Supplementary figure 5. Schematic drawing of the hairpin expression cassette. shRNAs are produced from the U6/tethracyclin-inducible (TetO) promoter. The U6 promoter can transcribe short RNAs by RNA Polymerase III. This expression cassette also contains genes coding for mCherry and Puromycine separated by a linker encoding the 2A self-cleaving sequence under the control of the EF1a promoter. Abbreviations: pA, polyadenylation signal; puro, puromycin; Ψ, packaging signal; T, terminator.
