# Supplementary material for: Flow cytometry-based functional selection of RNA interference triggers for efficient epi-allelic analysis of therapeutic targets
Source: BMC Biotechnol. 2014 Jun 21;14:57. doi: 10.1186/1472-6750-14-57 (PMC4074332; doi:10.1186/1472-6750-14-57)
Supplement: Additional file 6: Figure S6 — Sequences that have been scored as hairpins in screen. Lowercase letters signify hairpin loop. [file 1472-6750-14-57-S6.pdf]

Number of hits in screen	Sequence
3462	ACACATGTAGTTGTAGTGGATGGTGGTcatctctggatccagcagACCACCATCCACTACAACTACATGTGT
1959	GCGCTGCTCAGATAGCGATGGTCTGGatctctggatccagcagGGTGACCATCGCTATCTGAGCAGCGCT
486	ACTGTTGAATTTTCTCTAACTTCAAGGatctctggatccagcagCCTTGAAGTTAGAGAAAATTCAACAGT
163	AGTGGAGAATGTCAGTCTGAGTCAGGCatctctggatccagcaGCCTGACTCAGACTGACATTCTCCACT
113	ACCTTCGGTCTCATCTCTGGATCCAGCCatctctggatccagcaGGCTGGATCCAGAGATGAGACCGAAGGT
91	AGTGAGGGACAGCTTCCCTGGTTAGTCatctctggatccagcaGACTAACCAGGGAAGCTGTCCCTCACT
37	CCAGGACTTCCATTTGCTTTGTCCCGatctctggatccagcagCGGGACAAAGCAAATGGAAGTCCTGG
32	ACACATGTAGTTGTAGTGGATGGTGGTACCACCATCCACTACAACTACATGTGTA
32	GGGTCTAGCGGGATCCACTAGTAACGGatctctggatccagcagCCGTTACTAGTGGATCCCGCTAGAC
30	TCGAGATGTTCCGAGAGCTGAATcatctctggatccagcagCCTCATTCAGCTCTCGGAACATCTCGA
19	CCCGGGGCTCCACTGAACAAGTTGatctctggatccagcagcCAACTTGTTCAGTGGAGCCCCGGG
18	TGTGATGATGGTGAGGATGGGCCTCCcatctcggatccagcagcGGAGGCCCATCCTCACCATCATCACA
15	CCTGCATGGGCGGCATGAACCGGAGGatctctggatccagcagCCTCCGGTTCATGCCGCCCATGCAGGA
14	GTTAGAGAAAATTCAACAGCGAGGGACatctctggatccagcagGTCCCTCACTGTTGAATTTTCTCTAAC
10	ATTGGAAGTGGTTGCAATCTGGATTCAGCgaccagGCTGAATCCAGATTGCAACCACTTCCAAT
8	ATGAGACCTTCGGTCTCATCTCTGGAcatctctggatccagcagaTCCAGAGATGAGACCGAAGGTCTCAT
7	CTCACAATTCTGGAGTCTCTGGATCCCaGGGATCCAGAGACTCCAGAATTGTGAG
7	CTGGGAAGGGACAGAAGATGACAGGGGatctctggatccagcagCCCCTGTCATCTTCTGTCCCTTCCCAG
6	ACATTATTTCATTAACCCTCACAATcatctctggatccagcagATTGTGAGGGTTAATGAAATAATGTA
6	CCACTCGGATAAGATGCTGAGGAGGGGatctctggatccagcagCCCCTCCTCAGCATCTCATCCGAGTGG
6	CCATGTAATAAAAGGTGGTTTCAAGGCatctctggatccagcagCCTTGAAACCACCTTTTATTACATGG
5	CCCTTCAGATCCGTGGGCGTGAGCGatctctggatccagcagCGCTCACGCCCACGGATCTGAAGGG
3	GACAGCCAAGTCTGTGACTTGCACGTcatctctggatccagcagACGTGCAAGTCACAGACTTGGCTG
3	GTTCACAAGCAGGTGGTTGAGAGTGCTTAgaccagTAAGCACTCTCAACCACCTGCTTGTGAAC
2	TACAATCAGCCACATTCTAGGTAGGGGCatctctggatccagcaGCCCCTACCTAGAATGTGGCTGATTGTA
2	GTCGAGCGGCCGCCGGTGTGATGGATCatctctggatccagcagATATCCATCACACTGGCGGCCGCTCGA
2	TGAGACCTTCGGTCTCATCTggatccagcaggctccagagatgAGACCGAAGGTCTC
2	TGATGGTGAGGATGGGatctctggatccagcagCCCATCCTCACCATCATCACACTGGAA
2	TGGGAGCGTTCATCTGGACCTAACAATCatctctggataccagcaGATTGTTAGGTCCAGATGAAGCTCCCA
2	TTACGGTCTCATCTCTGGACCcagcatctctGGATCCAGAGATGAGACCGAA
1	AAACTCATGTTCAAGACAGAAGGGatctctggatccagcagCCCTTCTGTCTTGAACATGAGTTTCTTA
1	GTGACTGCTTGTAGATGGCCATGGCatctctggatccagcagGCCATGGCCATCTACAAGCAGTCACA

Supplementary figure 6. Sequences that have been scored as hairpins in screen. Lowercase letters signify hairpin loop.
