# Supplementary material for: Flow cytometry-based functional selection of RNA interference triggers for efficient epi-allelic analysis of therapeutic targets
Source: BMC Biotechnol. 2014 Jun 21;14:57. doi: 10.1186/1472-6750-14-57 (PMC4074332; doi:10.1186/1472-6750-14-57)
Supplement: Additional file 7: Figure S7 — Epiallelic HUVE cells comprising p53-targeting shRNA’s show graded p53 expression levels by immunoblotting. [file 1472-6750-14-57-S7.pdf]

Supplementary figure 7

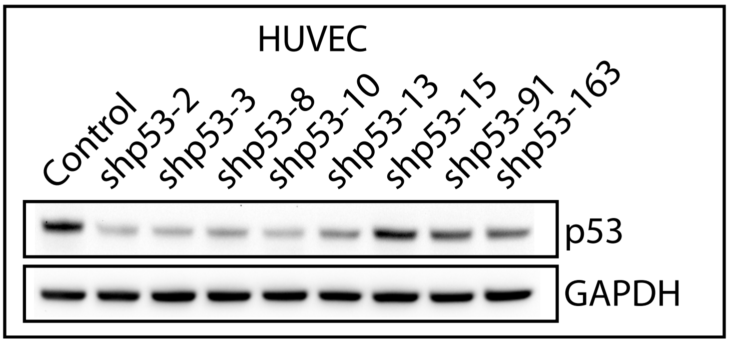

Epiallelic HUVE cells comprising p53-targeting shRNA's show graded p53 expression levels by immunoblotting.
